# Dasatinib attenuates airway inflammation of asthma exacerbation in mice induced by house dust mites and dsRNA

**DOI:** 10.1016/j.bbrep.2022.101402

**Published:** 2022-12-02

**Authors:** Yuki Nishimoto, Daiki Ando, Kosuke Irie, Ikumi Kainuma, Yuki Katayama, Shiori Sato, Tomohiro Suzuki, Mai Harada, Tsubasa Yoshida, Kazuhiro Ito, Yasuo Kizawa

**Affiliations:** a Laboratory of Physiology and Anatomy, School of Pharmacy, Nihon University, 7-7-1 Narashinodai, Funabashi, Chiba, 274-8555, Japan; b National Heart and Lung Institute, Imperial College London, Dovehouse Street, London, SW3 6LY, United Kingdom

**Keywords:** Src family kinase, Corticosteroid resistance, Asthma, Exacerbation

## Abstract

Asthma exacerbation is a significant clinical problem that causes resistance to corticosteroid therapy and elevated hospitalization risk. Src family kinases (SFKs) contribute to various steps of the immune response, such as airway inflammation in viral or bacterial infections and allergic asthma. Therefore, we determined the effects of dasatinib (DAS), a typical Src inhibitor, on a murine asthma exacerbation model induced by house dust mites (HDM) and synthetic analog of double-stranded RNA, poly(I:C). A/J mice were sensitized to intrapreneurial HDM twice every seven days and challenged with intranasal HDM once every second day for a total of six exposures, and/or exposed to poly(I:C) twice daily for three consecutive days. Drug treatments were performed twice daily for three days, starting one day after the last HDM challenge or 2 h before each poly(I:C) exposure. DAS improved poly(I:C)-induced acute inflammation dose-dependently. Both DAS and fluticasone propionate (FP) attenuated HDM-induced allergic airway inflammation. However, in HDM and poly(I:C) induced-asthma exacerbated mice, DAS significantly improved inflammatory cells in bronchoalveolar lavage fluid and histological changes in the lungs, whereas FP did not. Therefore, SFKs are important targets for controlling severe asthma refractory to conventional therapies.

## Highlights

• Asthma exacerbation is one of the important clinical problems which causes resistance to corticosteroids therapy.
• Dasatinib improved airway inflammation in a murine model of asthma exacerbation induced by poly(I:C) and house dust mite.
• The murine asthma exacerbation model in this study is refractory to corticosteroid therapy.

## 1 Introduction

Corticosteroid-insensitive airway inflammation is an important clinical problem in some patients with severe asthma. Asthma is one of the most common chronic inflammatory airway diseases, and inhaled corticosteroids (ICS) are commonly used in stepwise therapy based on severity and control assessments [1,2]. However, approximately 10% of asthmatics require the maximal dose of ICS, and some patients are refractory to corticosteroids [3]. Moreover, respiratory viruses and bacteria, such as respiratory syncytial virus, influenza virus, human rhinovirus, *Haemophilus influenzae*, and *Streptococcus pneumoniae*, often cause complications of airway infections in patients with asthma, resulting in asthma exacerbation and increasing the hospitalization risk of these patients [[4], [5], [6]]. These viral and bacterial infections can also cause a decrease in corticosteroid responsiveness *in vivo* and *in vitro* [[7], [8], [9]]. Additionally, we reported that repeated exposure to lipopolysaccharide (LPS), bacterial endotoxin, or polyinosinic-polycytidylic acid [poly(I:C)], a synthetic analog of double-stranded RNA (dsRNA), caused corticosteroid-insensitive inflammation in murine airways [10,11]. LPS and poly(I:C) are well-known ligand for toll like receptor (TLR) 4 and TLR3 which cause inflammatory responses similarly to bacterial and viral infections, respectively. Thus, the exacerbation of airway infection may be an important factor for corticosteroid resistance in asthma patients.

Src family kinases (SFKs) are non-receptor tyrosine kinases comprising nine members: Src, Lyn, Hck, Fgr, Fyn, Yes, Lck, Yrk, and Blk, which play key roles in regulating various cell functions, such as cell survive, growth, shape, differentiation, and migration [12,13]. In addition, SFKs are important for regulation of the immune system. Allergens activate the epidermal growth factor receptor (EGFR) via the activation of SKFs [14,15]. SFKs are also involved in generating reactive oxidant species (ROS) and activating NF-κB and signal transducer and activator of transcription (STAT) 1 via the TLRs signaling pathway [[16], [17], [18]]. We previously reported that a typical Src inhibitor, dasatinib (DAS), attenuated airway inflammation caused by repeated doses of LPS [19]. However, the effects of Src inhibitors on murine asthma exacerbation involving corticosteroid resistance is unclear. Therefore, in this study, we determined the effects of DAS on murine models of airway inflammation induced by house dust mites (HDM) and poly(I:C).

## 2 Materials and methods

### 2.1 Animal models

Five-week-old specific pathogen-free mice (male, A/J strain) were purchased from Sankyo Labo Service Co. Inc. (Tokyo, Japan). To induce allergic airway inflammation, mice were sensitized and challenged with the same amount of HDM with a previous study by Ogawa *et al* [20]. Mice were intraperitoneally administered 10 μg/animal of HDM (crude extract of *Dermatophagoides pteronyssinus*; Biostir Inc., Osaka, Japan) with 10 mg/mL of aluminum hydrate (Alum; Sigma-Aldrich, St. Louis, MO, USA) in phosphate-buffered saline (PBS) twice every 7 days and intranasally challenged with 100 μg/animal of HDM (40 μL/animal) in PBS once every other day for 11 days. To induce asthma exacerbation or acute inflammation, mice were anesthetized with 3% isoflurane and intranasally administered with 40 μL/animal of poly(I:C) (1 mg/mL, Sigma-Aldrich) twice daily for 3 consecutive days. The mice were intranasally treated with 35 μL/animal of vehicle [10% dimethyl sulfoxide (DMSO); Sigma-Aldrich], DAS (4–400 μg/mL; Cayman Chemical, Ann Arbor, MI, USA), or fluticasone propionate (FP; 50 μg/mL; Sigma-Aldrich), a typical steroidal anti-inflammatory drug, twice daily for 3 consecutive days from the next day after the last HDM exposure or 2 h prior to each poly(I:C) administration. In clinical practice, patients of airway viral infection will be treated with therapeutic agents at the time of infections, and patients of asthma will be treated in onset of allergic symptoms which will appear after the allergen exposure. Thus, in this study, mice were treated with drugs on the time points described above. DAS is clinically used as a BCR/Abl inhibitor that is prescribed for chronic myeloid leukemia and Philadelphia chromosome-positive acute lymphoblastic leukemia, and is the most clinically studied Src inhibitor [21]. Furthermore, oral administration of DAS reduced tumor necrosis factor-α (TNF-α) levels in the lung induced by intraperitoneal administration of LPS [22]. In this study, we used a presumably comfortable dose of DAS for intranasal treatment referencing the effective dose of oral therapy and the distribution in rat lungs [23]. In addition, in our previous study, 50 μg/mL of FP had enough suppressive effects on murine airway inflammation induced by one day exposure of LPS, but had no effects on it induced by three days exposure [10]. All animal procedures were performed following the guidelines of the Nihon University Animal Care and Use Committee (AP20PHA20-1). Mice were housed in a temperature and humidity controlled room (24 °C ± 1 °C and 55% ± 5% humidity) on a 12 h day–night cycle and fed with tap water and standard chow *ad libitum*.

### 2.2 Bronchoalveolar lavage fluid (BALF)

On the designated time points, under anesthesia, a cannula was placed into the murine trachea, BALF was collected by flushing three times with 0.1 mL/g body weight of saline. Lung tissue samples were collected for histological analysis. The BALF was centrifuged (500×*g*, 4 °C, 10 min) to remove total alveolar cells. Erythrocytes in the BALF cells were hemolyzed by resuspended on in 0.2% NaCl, and then, an equal amount of 1.6% NaCl was added. The cell suspension was used to determine the total cell count and cell composition in BALF.

### 2.3 Flow cytometry and enzyme-linked immunosorbent assay (ELISA)

FITC-conjugated anti-neutrophil antibody (clone 7/4, Acris Antibodies GmbH, Herford, Germany), PE rat anti-mouse Siglec-F (clone E50-2440, BD Pharmingen, Franklin Lakes, NJ, USA), FITC hamster anti-mouse CD11c (clone HL3, BD Pharmingen), monoclonal antibody against macrophages/monocytes-FITC (clone MOMA-2; Acris Antibodies), and propidium iodide (Sigma-Aldrich) were added to the BALF cell suspension for the detection of neutrophils, eosinophils, and macrophages. The cell composition in BALF was determined by using a flow cytometer (CytoFLEX; Beckman Coulter, Tokyo, Japan).

C-X-C motif chemokine ligand 1 (CXCL1), TNF-α, and interleukin (IL)-13 concentrations in the supernatants were detected by using Quantikine ELISA kits (R&D Systems Inc., Minneapolis, MN, USA) specific to each mediator.

### 2.4 Histological examination

Lung tissues were inflated with 10% phosphate-buffered formalin at the pressure at the height of approximately 20 cm liquid level for 4 h and then immersed in 10% phosphate-buffered formalin for tissue fixation for totally 72 h, followed by embedding in paraffin. Lung sections (4-μm thick) were cut from each lung, stained with hematoxylin and eosin (H&E), and observed under a light microscope.

### 2.5 Statistical analysis

Statistical analysis was performed using PRISM9 software (GraphPad Software Inc., CA, USA). Differences in multiple comparisons were assessed by analysis of variance followed by Dunnett's multiple comparison test, and unpaired t-tests assessed differences between two groups with Welch's correction. A *P*-value of <0.05 was considered statistically significant.

## 3 Results

### 3.1 Effects of dasatinib on a poly(I:C)-induced murine model of acute airway inflammation

**Fig. 1:**
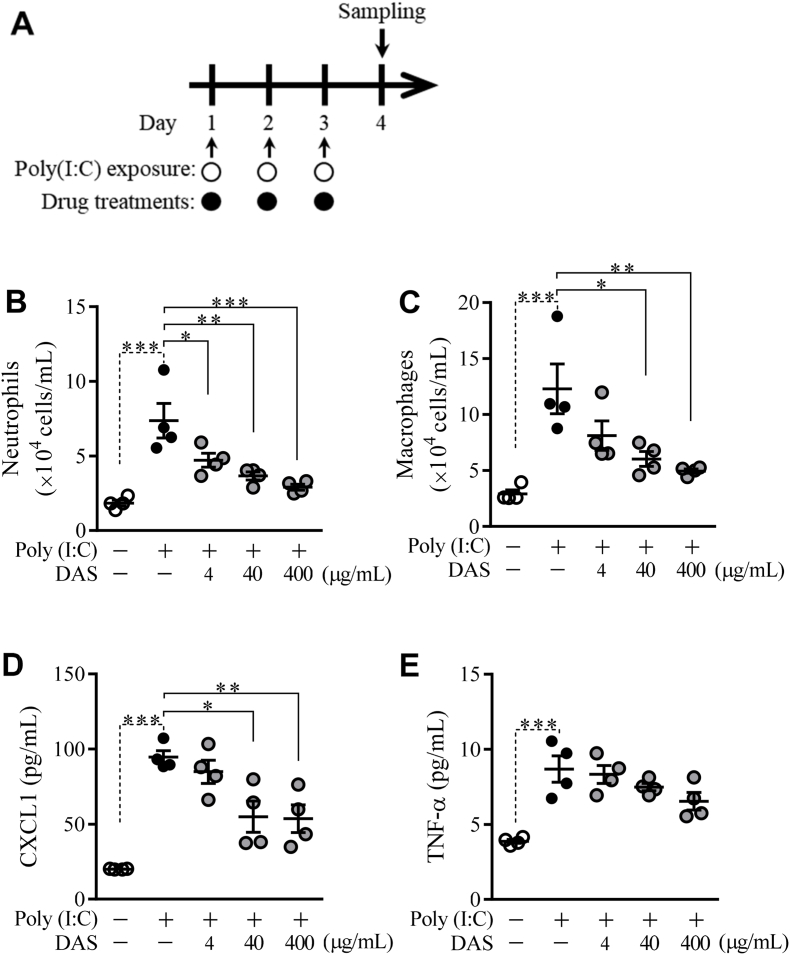
Dose-dependent improvement by DAS in acute airway inflammation induced by poly(I:C) Mice were intranasally exposed to poly(I:C) and treated with DAS according to the indicated scheme (A). The effects of DAS on the number of neutrophils (B) and macrophages (C), and the accumulation of CXCL1 (D) and TNF-α (E) in BALF were evaluated. Each horizontal bar shows the mean with the standard error (*P < 0.05; **P < 0.01; ***P < 0.001).

To evaluate the effects of DAS in the dose ranges of 4–400 μg/mL on murine acute airway inflammation, mice were exposed to poly(I:C) twice daily according to the indicated scheme (Fig. 1A). The BALF cell counts of neutrophils and macrophages were statistically significantly increased by poly(I:C). DAS showed a dose-dependent improvement in airway inflammation (Fig. 1B and C). More than low and medium doses of DAS are statistically significantly attenuated the increases in neutrophils and macrophages, respectively. Additionally, CXCL1 and TNF-α in the BALF were significantly induced by poly(I:C). DAS reduced the CXCL1 level, where the effects of medium and high doses were statistically significant, and slightly reduced the TNF-α level (Fig. 1D and E). These data suggest that medium and high-dose DAS significantly suppressed the acute airway inflammation induced by poly(I:C).

### 3.2 Effects of dasatinib on murine asthma induced by HDM

**Fig. 2:**
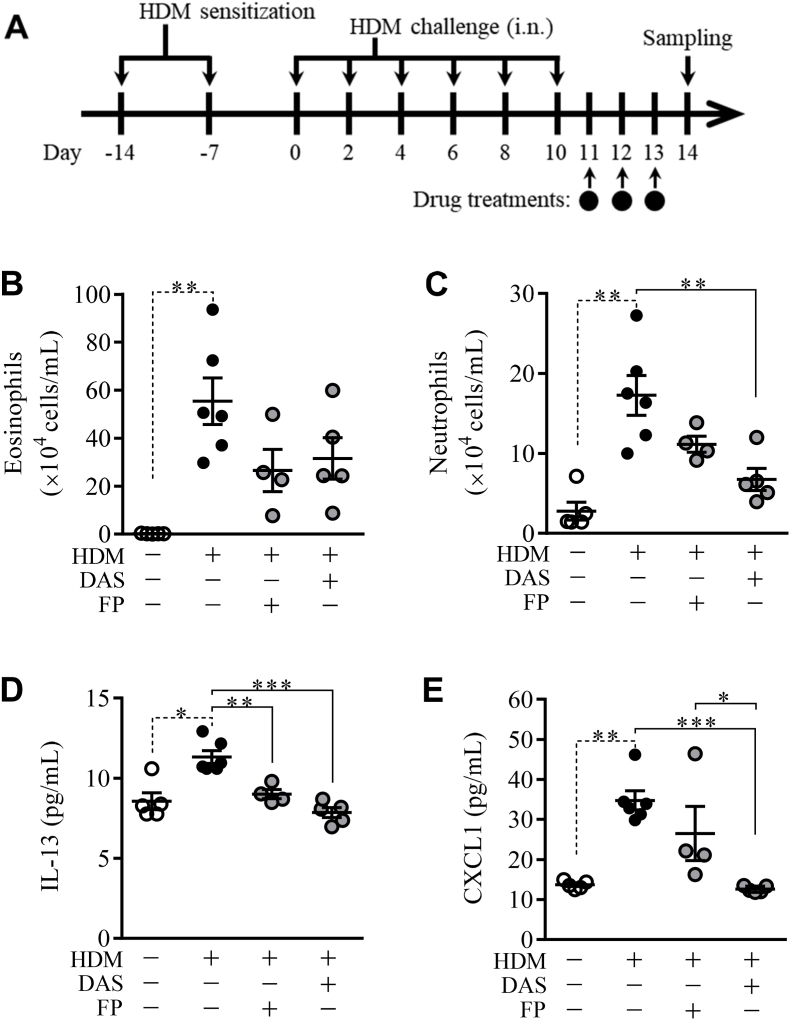
Anti-inflammatory effects of DAS and FP on HDM-induced allergic airway inflammation Mice were administrated with HDM and drugs according to the indicated scheme (A). The effects of DAS (40 μg/mL) were compared with those of FP (50 μg/mL) on the number of eosinophils (B) and neutrophils (C) and the accumulation of IL-13 (D) and CXCL1 (E). Each horizontal bar shows mean with standard error (*P < 0.05; **P < 0.01; ***P < 0.001).

Recently, SFKs have been reported to be involved in the inflammatory responses in murine allergic inflammation [14,15]. To determine the effects of DAS on murine asthma, mice were sensitized and challenged to HDM and treated with DAS (40 μg/mL) or FP (50 μg/mL) (Fig. 2A). The numbers of eosinophils and neutrophils were statistically significantly increased by HDM (Fig. 2B and C). FP slightly reduced the increase in the number of eosinophils and neutrophils in the BALF. DAS reduced the number of eosinophils slightly and neutrophils significantly (Fig. 2B and C). Furthermore, DAS significantly reduced both IL-13 and CXCL1 levels, and FP reduced IL-13 levels (Fig. 2D and E).

### 3.3 Effects of dasatinib on asthma exacerbation induced by poly(I:C) and HDM

**Fig. 3:**
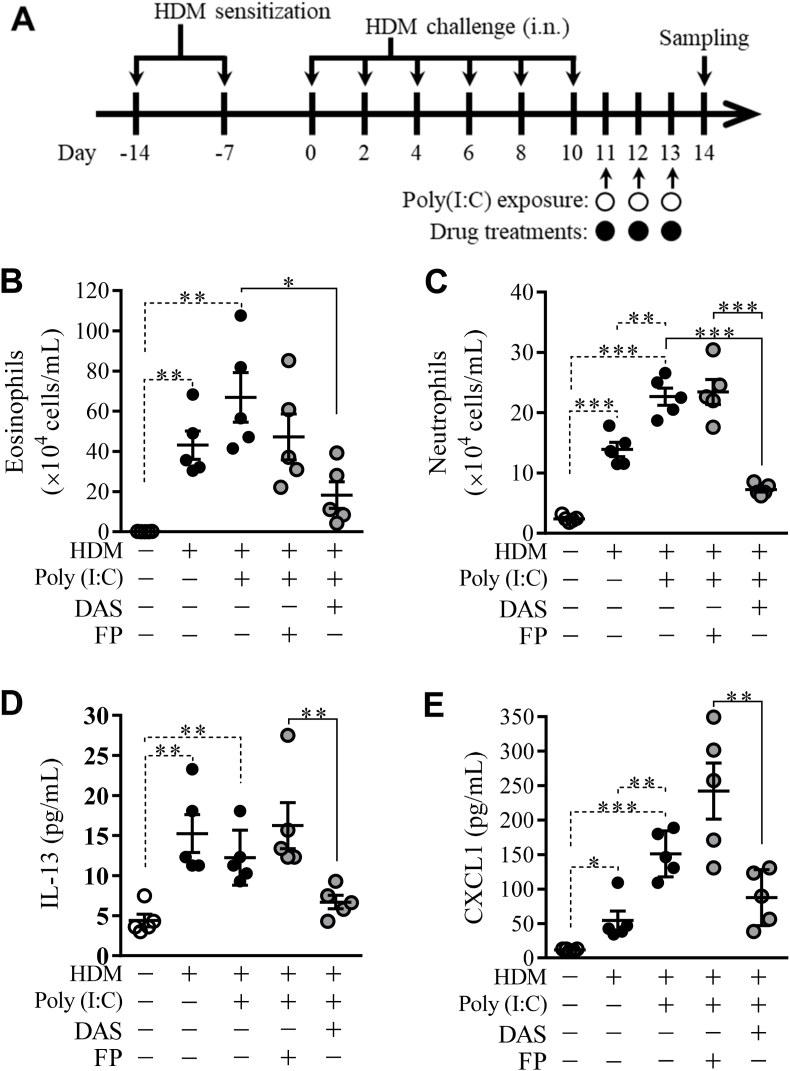
Improvement by DAS in asthma exacerbation induced by HDM and poly(I:C) Mice were administrated with HDM, poly(I:C), and drugs according to the indicated scheme (A). The effects of DAS (40 μg/mL) were compared with those of FP (50 μg/mL) on the number of eosinophils (B) and neutrophils (C) and the accumulation of IL-13 (D) and CXCL1 (E). Each horizontal bar shows mean and standard error (*P < 0.05; **P < 0.01; ***P < 0.001).

**Fig. 4:**
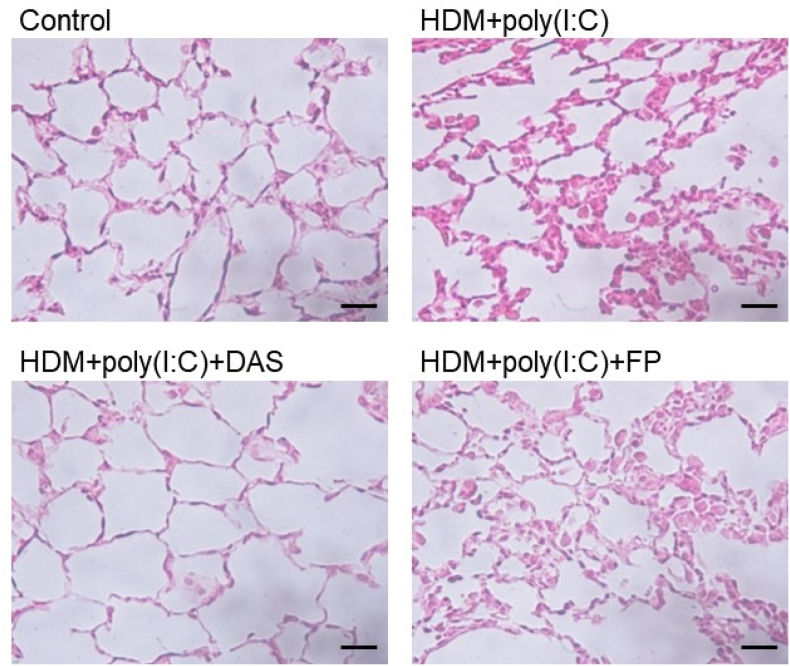
Effects of DAS on HDM and poly(I:C)-induced histological changes in murine lungs refractory to intranasal corticosteroid Lung sections (4-μm thick) obtained from the control, HDM + poly(I:C), HDM + poly(I:C) +DAS, and HDM + poly(I:C)+FP groups were stained with H&E staining for histological observation. Original magnification: 400 × . Scale bar represents 25 μm.

Previously, we reported that repeated-dose poly(I:C) caused corticosteroid-insensitive airway inflammation in mice. Thus, to evaluate asthma exacerbation, mice which sensitized and challenged with HDM were exposed to poly(I:C) twice daily according to the indicated scheme (Fig. 3A). HDM alone caused statistically significant increases in eosinophil and neutrophil counts. Poly(I:C) caused a significant increase in neutrophils compared to HDM alone (Fig. 3B and C). In HDM- and poly(I:C)-exposed mice, FP slightly suppressed the increase in eosinophils and had no effect on neutrophils. In contrast, DAS significantly attenuated the increase in eosinophil and neutrophil numbers (Fig. 3B and C). The similar suppressive effects were also observed in the accumulation of IL-13 and CXCL1 in the BALF. HDM alone caused statistically significant increases in BALF levels of IL-13 and CXCL1. Poly(I:C) increased the CXCL1 level but did not affect the IL-13 level induced by HDM alone (Fig. 3D and E). DAS slightly decreased the cytokine/chemokine levels, although FP did not decrease (Fig. 3D and E). Moreover, HDM and poly(I:C) caused the accumulation of inflammatory cells in the lung tissue. FP did not result in histological changes in the lung. However, DAS improved the inflammatory changes in the lungs (Fig. 4).

## 4 Discussion

Corticosteroid-insensitive inflammation is an important barrier to controlling the symptoms of severe asthma [3]. Moreover, viral or bacterial infections can reduce the responsiveness to corticosteroids [[7], [8], [9], [10], [11]]. Previously, we reported that the same dose range of DAS suppressed LPS-induced airway inflammation in a dose-dependent manner [19]. Thus, pharmacological control of corticosteroid-insensitive inflammation is important for treating pulmonary diseases. Thus, we examined the effects of DAS on three murine models of airway inflammation. DAS attenuated poly(I:C)-induced acute and HDM-induced allergic airway inflammation. And additional exposure to poly(I:C) on HDM-induced asthmatic mice caused exacerbation of airway inflammation and corticosteroid insensitivity. DAS also improved corticosteroid insensitive asthma exacerbation induced by HDM and poly(I:C).

TLR3 localizes in endosomes and is activated by dsRNA. dsRNA-activated TLR3 triggers downstream signaling pathways via phosphorylation of TLR3 by EGFR, Src, and Bruton's tyrosine kinase (Btk). Then, interferon regulatory factor 3 and NF-κB [24] are activated. In a previous study, murine acute airway inflammation induced by 3 days exposure to poly(I:C) involved airway hyperresponsiveness and corticosteroid insensitivity [11]. Thus, this murine models of acute inflammation might mimic, at least in part, airway virus infections. And in this models, 5–500 μg/mL of FP had no suppressive effects on murine airway inflammation caused by repeated poly(I:C) exposure for 3 days [11]. In contrast, DAS improved poly(I:C)-induced acute inflammation in a dose-dependent manner. DAS inhibits Src and Btk kinases [25,26]. Therefore, the inhibitory effects of DAS on SFKs contribute to the improvement in corticosteroid-refractory inflammation caused by poly(I; C).

HDM is a common perennial allergen for human atopic diseases, which is typical in 50%–80% of allergic asthmatics [27]. *D. pteronyssinus* and *D. farina* are major indoor HDMs, whose antigenicity comes from proteases in HDM itself and their feces and bacterial and fungal products [27,28]. HDM also causes allergic airway inflammation and accumulation of various cytokines in mice [20], and in this study, mice have responded in similar manner to the finding of Ogawa et al. Various proteases are classified as HDM allergens, which comprise papain-like cysteine, trypsin-like serine, chymotrypsin-like serine, and collagenolytic-like serine proteases [28]. These proteases deliver allergens in the airway lumen by cleaving intracellular tight junctions, and HDM allergens upregulate the expression of IL-25, IL-33, and thymic stromal lymphopoietin and activate type 2 innate lymphoid cells (ILC2) in the lung mucosa [27,28]. Interestingly, TLR4 deficient (TLR4^-/-^) and wild-type dendritic cell-received TLR4^-/-^ chimeric mice are unresponsive to HDM [29]. This report suggests that TLR4 on structural cells triggers an immune response against HDM. HDM activates ILC2 responses via dual oxidase 1 (DUOX1)-dependent Src/EGFR activation and airway epithelial IL-33 production [14,30]. SFK contributes to TLR4 signaling pathway regulation. Lyn, one of SFKs, positively regulates the TLR4/TRAF6 pathway and negatively regulates the TLR4/PI3K pathways [31]. Additionally, LPS induces NF-κB activation and intercellular adhesion molecule-1 (ICAM-1) expression via the activation of TLR4/Src/EGFR signaling pathway in human airway epithelial cells [16]. Conversely, T-cell receptor (TCR) and IgE induce allergic responses via EGFR and Src activation [15], and the TCR signaling pathway is also associated with TLR2, 5, and 7, Lck, and Fyn [32,33]. DAS also inhibits Lyn and Fyn, other kinases of SFKs, in addition to Src [34], and we have also demonstrated that DAS improved murine allergic inflammation in the airway. Therefore, SFK inhibitors might potently control airway inflammation in asthma and airway infection. We also showed an improvement in airway inflammation by DAS in a murine asthma exacerbation model caused by both HDM and poly(I:C) exposure. In this study, exposure to poly(I:C) resulted in amplifying airway inflammation in mice induced by HDM, and FP had little suppressive effect on inflammation. The differences in the CXCL1 level and the neutrophil count between HDM + poly(I:C) and HDM group in Fig. 3 were the similar amount with poly(I:C) exposed mice observed in Fig. 1. And our previous study indicated that repeat-dosed poly(I:C) caused corticosteroid-insensitive airway inflammation [11]. Thus, this study indicates that inhibitors against SFKs may be potent anti-inflammatory drugs to control asthma exacerbation by modulating asthma symptoms and corticosteroid-insensitive inflammation in airway infections.

This study has some limitations. Poly(I:C) mimics the inflammatory responses in infections, unlike natural viral infections. However, the effects of DAS on viral clearance are unexplored. DAS has extensive inhibitory effects on various kinases. However, we did not evaluate intracellular kinase activity or related signaling pathways or whether Src inhibitory property had contributed the anti-inflammatory effects of DAS. Thus, further investigation is needed to clarify whether SFK inhibitors affect viral or bacterial clearance, which intracellular signaling pathway or SFK subtypes the inhibitory effects of DAS act through to improve asthma exacerbation, and whether intracellular kinases other than SKFs contribute to the effects of DAS.

In conclusion, we showed *in vivo* improvement by DAS in asthma exacerbation refractory to corticosteroid therapy. SFKs are potent and broad regulators of multiple parts of the immune response and various cellular functions. Thus, SFKs may be important targets for controlling severe asthma refractory to conventional therapies. Future research on the detailed mechanism of the anti-inflammatory effects of SFK inhibitors will contribute to developing novel therapies controlling intractable asthma.

## Declaration of competing interest

The authors declare that they have no known competing financial interests or personal relationships that could have appeared to influence the work reported in this paper.

## Data Availability

No data was used for the research described in the article.
